# Evidence of fructose metabolism in colorectal cancer

**DOI:** 10.1038/s41420-025-02745-w

**Published:** 2025-10-16

**Authors:** Giuseppe Sigismondo Sica, Julia Bischof, Lukas Funke, Juhl Hartmut, Eleonora Candi, Alessandro Mauriello, Manuel Scimeca, Xinyue Gao, Luca Savino, Francesca Servadei, Ruigang Yang, Lu Wang, Qing Zhao, Wen-Lian Chen, Qiang Sun, Wei Jia, Gerry Melino

**Affiliations:** 1 Department of Surgical Science, University of Rome Tor Vergata, Rome, Italy; 2 Indivumed GmbH, Falkenried, Hamburg, Germany; 3 Department of Experimental Medicine, TOR, University of Rome Tor Vergata, Rome, Italy; 4 Laboratory of Advanced Biotechnology, Beijing Institute of Biotechnology; Research Unit of Cell Death Mechanism, 2021RU008, Chinese Academy of Medical Science, Beijing, China; 5 Anatomic Pathology, Department of Integrated Care Processes, University of Rome Tor Vergata, Rome, Italy; 6 Department of Pharmacology and Pharmacy, University of Hong Kong, Hong Kong, China; 7 Center for Translational Medicine and Shanghai Key Laboratory of Diabetes Mellitus, Shanghai Sixth People’s Hospital Affiliated to Shanghai Jiao Tong University School of Medicine, Shanghai, China; 8 Cancer Institute, Longhua Hospital, Shanghai University of Traditional Chinese Medicine, Shanghai, China

**Keywords:** Colon cancer, Predictive markers

## Abstract

Colorectal cancer (CRC) represents a significant global health burden, contributing significantly to cancer-related mortality. The underlying genetic and metabolic underpinnings of CRC remain incompletely understood. In this study, we conducted a comprehensive investigation into metabolic perturbations in 29 CRC patients utilizing targeted omics approaches, compared to public databases. Additionally, we examined serum and tissue samples from 12 patients, with and without preoperative glucose challenge, using targeted metabolomics to investigate glucose and fructose metabolism. Notably, elevated levels of serum D-Fructose and L-Lactic acid following glucose administration indicate augmented glycolytic activity and polyol pathway-mediated glucose conversion (to fructose). Despite variations in tumor responses, our results underscore the potential significance of fructose metabolism in CRC progression, shedding light on therapeutic avenues targeting the Warburg effect. This research lays a solid foundation for future translational research into metabolic interventions in CRC treatment and enhances our understanding of cancer-related metabolic reprogramming.

## Introduction

Colorectal cancer (CRC) ranks among the most prevalent malignancies globally, with high morbidity and mortality [1]: 1.9 million new cases are diagnosed annually, accounting for 10% of all cancer-related deaths worldwide [2]. The pathogenesis of CRC is multifactorial, involving genetic, environmental, and metabolic factors. Among these, metabolic reprogramming, the alteration of cellular energy pathways to meet the demands of uncontrolled growth, is increasingly recognized as a hallmark of cancer [3]. Still, the underlying biochemical pathways regulating the CRC progression are still elusive. Specifically, does CRC exhibit the Warburg effect [4]? While much research has focused on glucose metabolism, emerging evidence suggests that fructose metabolism also plays a critical role in cancer progression [5]. A recent report suggests an association between dietary fructose and human colon DNA methylation, implying high CRC risk [5]. The authors identified 4263 right colon fructose-associated differentially methylated regions, compared to only 24 in matched, left colon. This indicates a striking association with fructose exposure, also confirmed in organoids from african-american individuals, supporting the idea that dietary fructose exerts a greater CRC risk-related effect in the right than left colon among adults, and potentially underlying nutritional and racial disparities in CRC incidence [5].

**Fig. 1:**
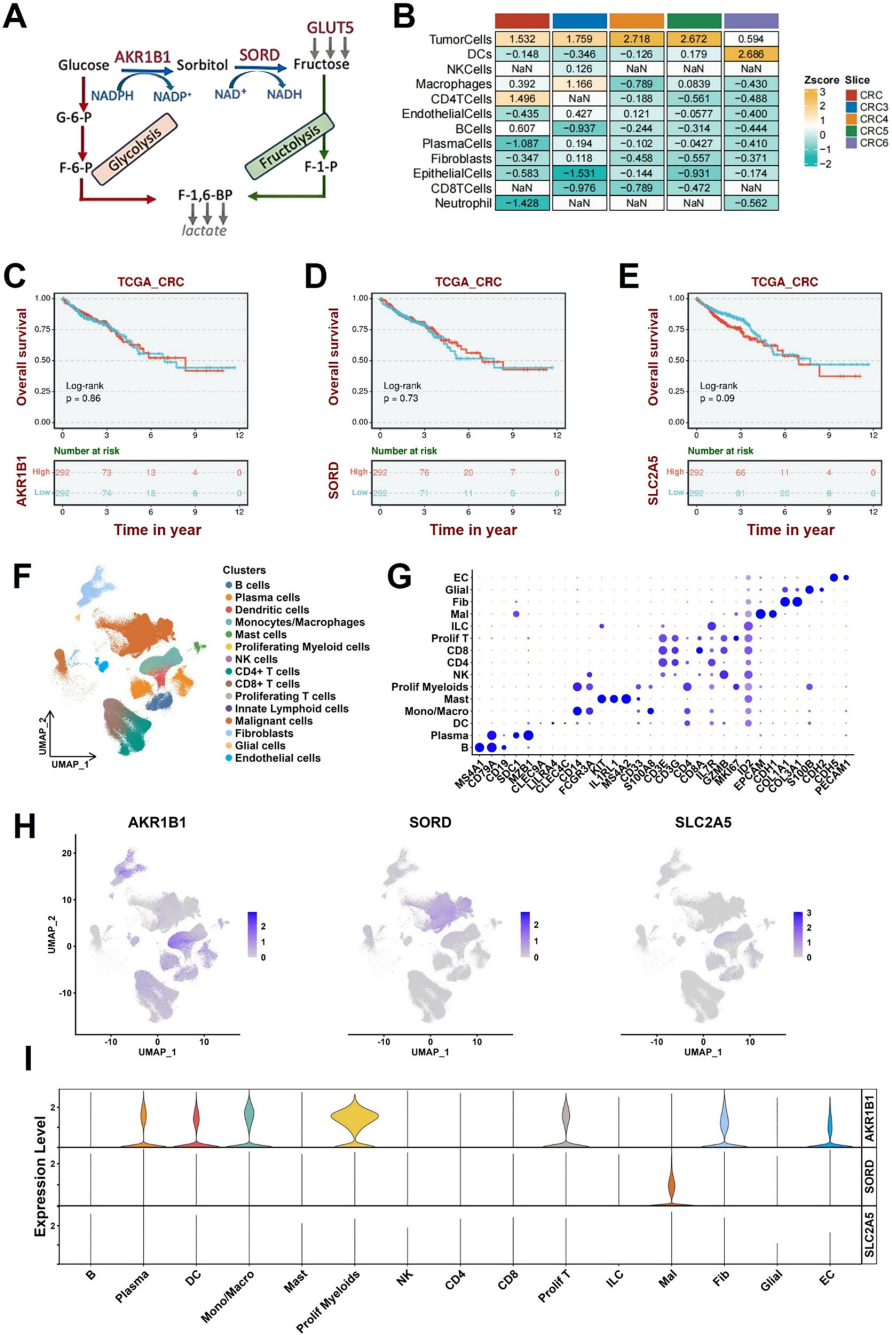
Metabolic pathway, expression analysis and spatial transcriptomic of SLC2A5, AKR1B1, and SORD in colon cancer. **A** Schematic representation of the glucose-to-fructose metabolic pathway highlighting the role of AKR1B1 in sorbitol synthesis and SORD in converting sorbitol to fructose. GLUT5 (encoded by the SLC2A5 gene) mediates fructose uptake. **B** Heat maps of SORD gene expression in different cell types in CRC spatial transcriptome slices, indicating a significant expression of SORD in tumour cells; CRC 1-3-4-5-6 indicates distinct slice of CRC. The colour of the heat map represents standardized z-score for gene expression level. Survival analysis of CRC patients with AKR1B1 (**C**), SORD (**D**) or SLC2A5 (**E**) expression by Kaplan–Meier analysis. The horizontal coordinate is survival time *t*, and the vertical coordinate is the probability of an individual surviving beyond time *t*. The red is the high expression group, and the blue is the low expression group. Log-rank test tests the difference in survival curves between the two groups, and *P* < 0.05 means significant. **F** UMAP visualization of 302,474 cells annotated by cell type identities. **G** Dot plot displaying lineage-specific marker gene expression. **H** Spatial co-expression patterns of target genes (*AKR1B1*, *SORD* and *SLC2A5*) across cell clusters, with **I** corresponding violin plots quantifying their expression distributions. Curves filled with the indicated colors represent cell proportion per population. Single Cell Transcriptomics for AKR1B1 (**A**), SORD (**B**) or SLC2A5 (**B**) in CRC samples, see also Supplementary Fig. S1 for more details.

We have recently performed a study on the Warburg effect, identifying the Polyol Pathway (PP) as a critical contributor that transforms glucose in fructose. Fructose, a monosaccharide increasingly abundant in modern diets, has been implicated in exacerbating tumorigenesis through its unique metabolic pathways [6, 7]. We found that tumor cells in vitro can transform glucose into fructose via the polyol pathway (PP) [2, 3], see Fig. 1A. Accordingly, aldose reductase (AKR1B1) is able to convert glucose to sorbitol using NADPH. In the second step of the PP, sorbitol leads to fructose formation by the enzymatic action of the sorbitol dehydrogenase (SORD), converting NAD+ into NADH. This alternative metabolic pathway diverts glycolysis to fructolysis, avoiding the regulated inhibition of Hexokinase (HK) and phosphofructokinase (PFK). We also found that some cancer cells, instead of generating endogenous fructose, as able to directly uptake exogenous fructose via the GLUT5 receptor [2, 8]. Thus, this pathway not only facilitates fructose production but also enhances glycolytic flux, promoting tumor growth and metastasis [9]. Accordingly, aldose reductase, the enzyme catalyzing the first step of the polyol pathway, has been found to be upregulated in various malignancies, further linking fructose metabolism to cancer progression [10].

Despite these insights, the interplay between glucose and fructose metabolism in CRC remains only partially understood. This study aims to address this gap by leveraging targeted metabolomics to analyze metabolic changes in CRC. By comparing serum and tissue samples from patients with and without glucose loading, we seek to elucidate the role of glucose-to-fructose conversion and its impact on CRC metabolism. Our findings have the potential to inform novel therapeutic strategies targeting metabolic vulnerabilities in CRC.

## Results

### Expression of AKR1B1, SORD and SLC2A5 in CRC patients

We have recently elucidated the roles of AKR1B1 and SORD in the conversion of endogenous fructose, along with GLUT5 (encoded by the SLC2A5 gene) in the uptake of exogenous fructose [2, 3]. These findings underscore the involvement of the polyol pathways in the Warburg effect observed in several cancers.

To investigate the potential impact of genes associated with the PP on CRC progression, a bioinformatic analysis, including spatial transcriptomics, was performed (Fig. 1B–I). Single-cell transcriptomic analysis conducted on five CRC cohorts showed that the SORD gene is highly expressed in tumor cells compared to stromal and inflammatory cells (Fig. 1B). No significant difference in overall survival was observed for AKR1B1 (Fig. 1C), SORD (Fig. 1D) and SLC2A5 (Fig. 1E). Single-cell transcriptomic analysis (Figs. 1F–I and S1A) revealed that AKR1B1 expression is higher in fibroblasts and inflammatory cells compared to colon cancer cells (Fig. 1H, I). Higher expression of SLC2A5 was observed in malignant cells (Fig. 1H, I and Supplementary Fig. S1). More detailed single cells transcriptomic analysis for AKR1B1, SORD and SLC2A5 are reported in supplementary Fig. S1 and 2. The expression of all investigated genes in relation to patient prognosis across several colon cancer cohorts is also reported in Supplementary Fig. S1D.

**Fig. 2:**
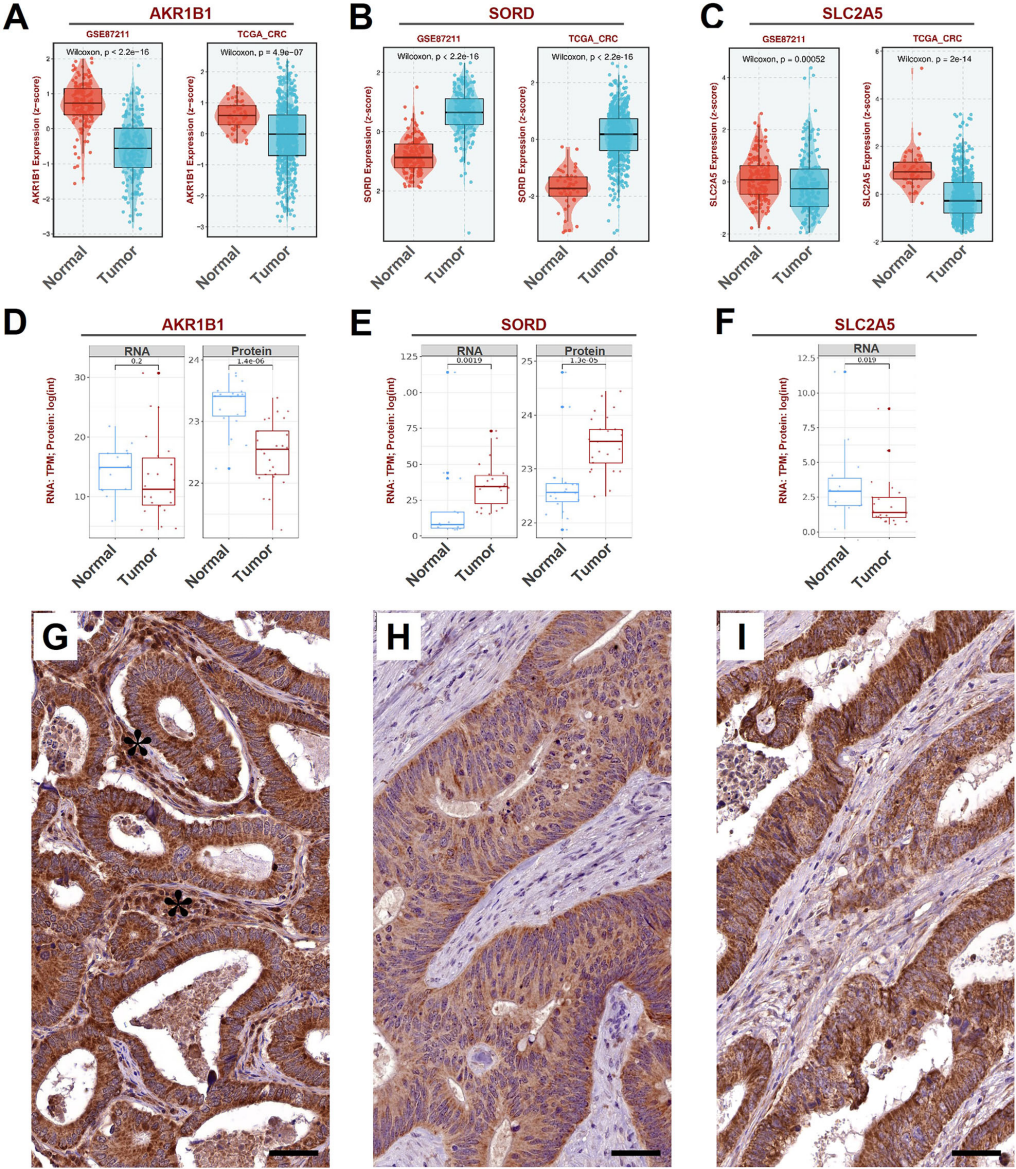
mRNA, protein, and immunohistochemical analysis in CRC patients. Differential expression analysis, in two distinct datasets of AKR1B1 (**A**), SORD (**B**) or SLC2A5 (**C**), and in tumour versus normal colon tissues. SLCL2A5 and AKR1B1 expression significant decrease in tumour tissues as compared to normal tissues; conversely, SORD expression significantly increases in tumours as compared to normal tissues. The expression levels of genes are standardized using z-score. Box plots comparing mRNA and protein (MS/MS) expression levels of AKR1B1 (**D**), SORD (**E**), and SLC2A5 (**F**), between normal and tumour tissues. Samples correspond to the patients indicated in Table 1. The values originated from public databases, **A**–**C**, (also see Supplementary Table I), fully matching the patients data. For technical problems, protein data were not available for GLUT5, codified by the *SLC2A5* gene. Immunohistochemistry was performed for AKR1B1 (**G**), SORD (**H**), and GLUT5 (**I**), from patients in Table 1. Numerous AKR1B1-positive cancer cells are observed in CRC samples from a patient (**G**); AKR1B1expression is also detected in stromal cells (asterisks) (**G**). Strong SORD positivity is observed in CRC patients, specifically in tumor cells (**H**). Numerous GLUT5-positive cancer cells are present in CRC samples (**I**). Scale bar: 100 µm.

Bioinformatic analysis also revealed distinct expression patterns of AKR1B1 (Fig. 2A), SORD (Fig. 2B), and SLC2A5 (Fig. 2C) in colon samples compared to tumor samples. Specifically, AKR1B1 and SLC2A5 exhibited significantly lower expression in CRCs compared to normal tissues, while SORD expression significantly increases in CRCs.

In addition to the analysis of public datasets, we conducted a direct investigation using samples collected from our hospital. The expression levels of SLC2A5, AKR1B1, and SORD were analyzed in our cohort comprising 29 CRC samples (Table 1) and 14 adjacent normal tissues using RNA sequencing (RNASeq) and proteomics. Table 1 reports the main clinical and molecular characteristics of enrolled patients. The molecular data from our samples mirrored those from the public investigated colon cancer cohort (Fig. 2A–C). Specifically, AKR1B1 exhibited comparable mRNA levels between tumors and normal tissues, yet the protein level was significantly reduced in tumors (*p* < 0.0001) (Fig. 2D). Conversely, both mRNA expression (*p* = 0.0019) and protein levels (*p* < 0.0001) of SORD were notably higher in CRCs than in adjacent normal tissues (Fig. 2E). SLC2A5 mRNA expression was lower in CRC patients compared to normal tissues (*p* = 0.019) (Fig. 2F). No proteomic data was available for SLC2A5.

In summary, our samples demonstrated the presence of all PP components, suggesting a potential contribution of these enzymes to the Warburg effect. However, distinct roles were observed for AKR1B1 and SORD despite their involvement in the same pathway.

### Immunohistochemistry

To further confirm the observed expression of PP enzymes at steady-state protein levels, we performed several immunohistochemical analyses for SLC2A5, AKR1B1, and SORD in the 12 CRC samples treated or not with a glucose load prior to surgery (Fig. 2G–I). All examined CRC samples exhibited significant expression levels of SLC2A5, AKR1B1, and SORD. The staining showed in all cases a significant expression in cancer cells, as compared to stromal tissue, further supporting the involvement of the PP enzymes in CRC samples.

### Serum metabolite profiles

To further investigate the impact of glucose loading on the PP, an observational prospective study was conducted. This study enrolled 12 patients diagnosed with gastrointestinal tumors, spanning various tumor sites including the hepatic flexure, sigma, rectum, recto-sigmoid junction, colon, and liver (Table 1). Patient recruitment was sequential and not based on other criteria. Inclusion criteria mandated histopathological confirmation of CRC and no prior chemotherapy or radiotherapy to minimize confounding factors.

**Fig. 3:**
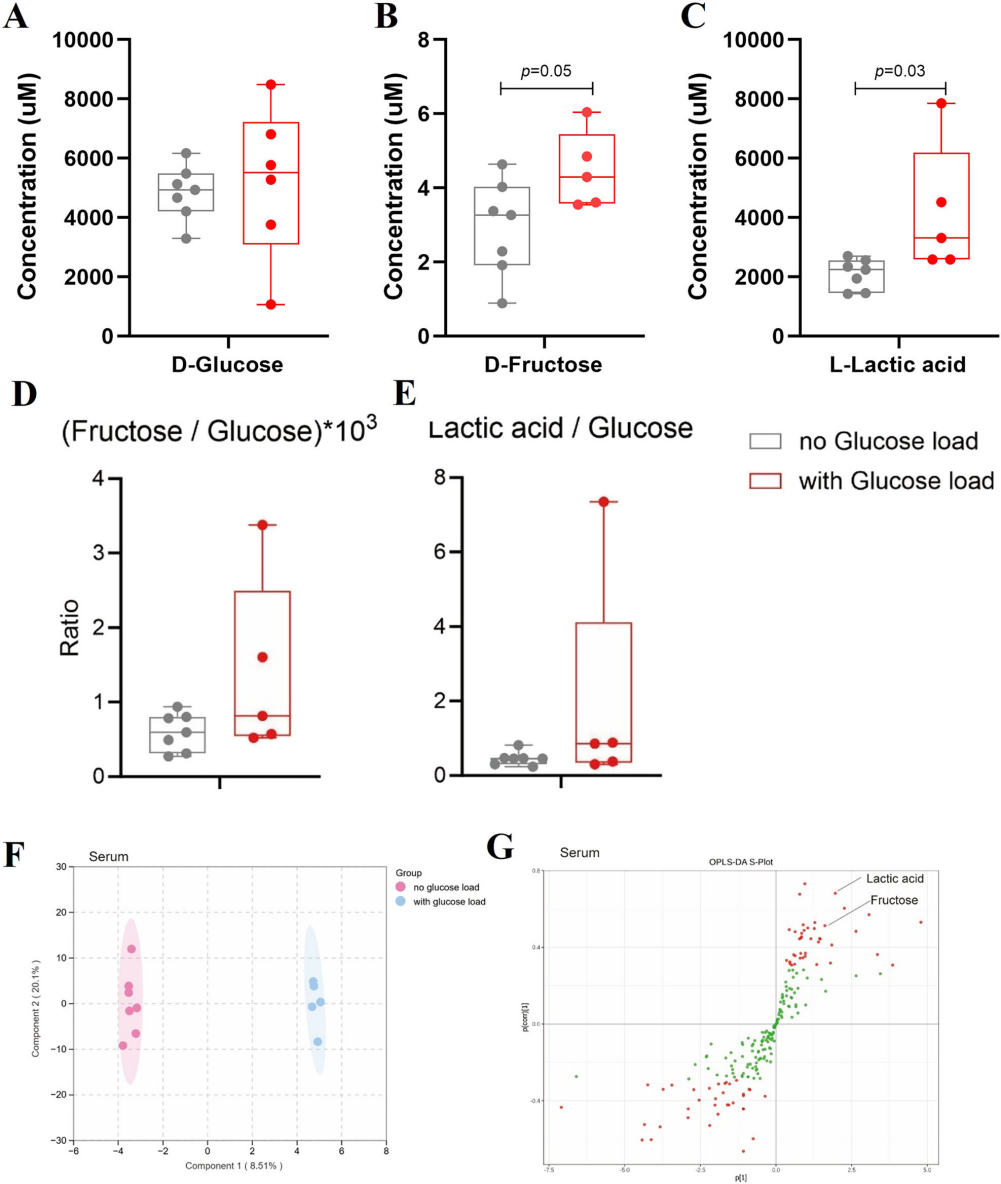
Serum metabolite profiles in glucose-loaded versus non-loaded groups. The concentration of D-Glucose (**A**), D-Fructose (**B**), and L-Lactic acid (**C**) in serum. Serum metabolite levels normalized to glucose concentration: **D** Fructose/Glucose ratio ×10³ and **E** Lactic acid/Glucose ratio. Normalization revealed high inter-individual variability in glucose handling. **F** OPLS-DA score plot showing distinct clustering of patients according to glucose loading status. **G** Corresponding S-plot identifies D-Fructose and L-Lactic acid as key contributors to group separation, with VIP > 1.

To elucidate the potential Warburg effect, patients were divided into two groups: one without glucose loading (*n* = 7) and another with glucose loading (*n* = 5). The glucose-loaded group underwent an oral glucose tolerance test (OGTT) one hour before surgery, corresponding to two hours before sample collection, aiming to boost the metabolic pathway and consequently enhance the Warburg effect. Serum and surgical samples were collected from patients who underwent glucose loading before surgery (*n* = 5) and those who did not (*n* = 7). D-Glucose levels did not exhibit a statistically significant difference between the serum samples of the non-glucose-loaded and glucose-loaded groups (Fig. 3A), indicating consistent glucose concentrations across conditions. Conversely, D-Fructose concentrations showed a trend towards higher levels in the glucose-loaded group compared to the non-loaded group (*p* = 0.05) (Fig. 3B). Moreover, both D-Fructose (Fig. 3B) and L-Lactic acid (Fig. 3C) concentrations were elevated in the glucose-loaded group compared to the non-loaded one (*p* = 0.05 and *p* = 0.03, respectively). These results suggest that glucose loading induces detectable metabolic alterations in circulation, reflecting the dynamic interplay between systemic and tumor-specific metabolism. The rise in lactate levels (Fig. 3C) indicates the presence of the Warburg effect, while the increase in fructose levels (Fig. 3B) suggests the involvement of the PP. To account for the observed inter-patient variability in serum glucose concentrations, we also normalized the levels of D-Fructose and L-Lactic acid to the corresponding D-Glucose concentration for each individual patient. As shown in Fig. 3D, E, while raw metabolite levels differed significantly between groups, the Fructose/Glucose and Lactate/Glucose ratios did not reach statistical significance. These results indicate that the individual variability in glucose uptake and metabolism may modify group-level differences when using absolute concentrations To further strengthen these findings, we performed a multivariate statistical analysis to assess the overall impact of glucose administration on the serum metabolomic profile (Fig. 3F). This analysis clearly separated glucose-loaded from non-loaded patients, supporting distinct metabolic phenotypes associated with glucose exposure. The corresponding S-plot (Fig. 3G) highlighted D-Fructose and L-Lactic acid as the most discriminative metabolites, with Variable Importance in Projection (VIP) scores exceeding 1. These metabolites were thus identified as major contributors to the group separation. This multivariate analysis confirms and expands upon the univariate results, supporting the robustness of our data and reinforcing the hypothesis that both glycolysis and the polyol pathway are upregulated upon glucose challenge.

Overall, these data support the presence of the Warburg effect in CRC patients, accompanied by activation of the PP.

### Metabolic alterations in tumor tissues

**Fig. 4:**
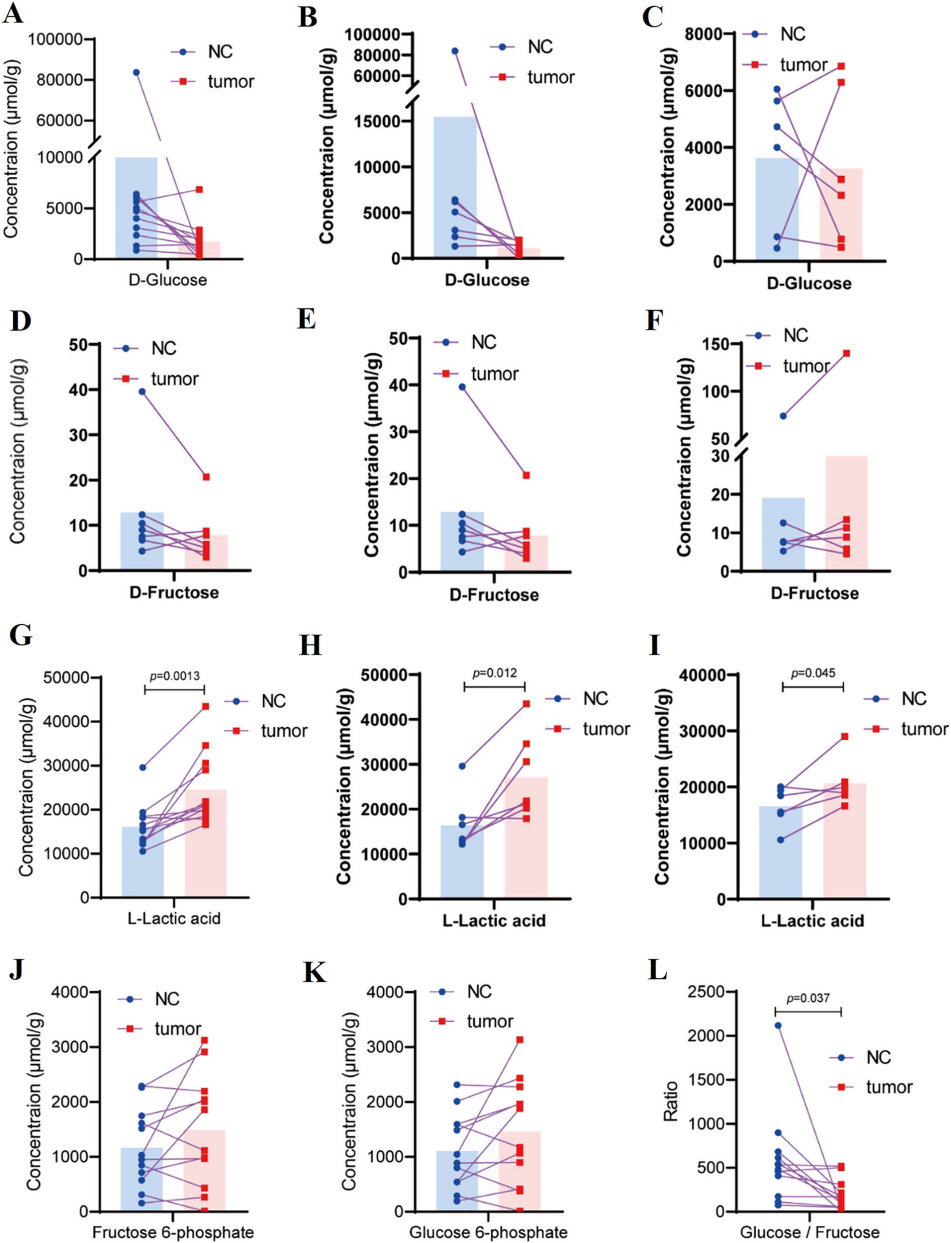
Metabolite concentrations (D-Glucose, D-Fructose, L-Lactic acid) in tumor and adjacent tissues. D-Glucose concentration in tumor and adjacent tissues across all collected samples (**A**), as well as in groups without (**B**) and with (**C**) glucose load. D-Fructose concentration in tumor and adjacent tissues across all collected samples (**D**), as well as in groups without (**E**) and with (**F**) glucose load. L-Lactic acid concentration in tumor and adjacent tissues across all collected samples (**G**), as well as in groups without (**H**) and with (**I**) glucose load. Fructose 6-phosphate(**J**), and Glucose 6-phosphate (**K**) in tumor and adjacent tissues across all collected samples. The ratio of glucose to fructose in tumor and adjacent tissues across all collected samples (**L**).

The analysis of metabolites comparing CRC samples to adjacent normal tissues unveiled significant alterations in glucose and fructose metabolism (Fig. 4). While no statistically significant differences were observed, D-Glucose concentrations were noted to be lower in tumor tissues compared to normal tissues, both across all patients (Fig. 4A) and in those who did not undergo glucose loading (Fig. 4B). Glucose loading before surgery appeared to normalize glucose levels among the experimental groups (Fig. 4C). A similar pattern was observed for D-Fructose levels (Fig. 4D–F). On the other hand, L-Lactic acid concentrations were significantly elevated in tumor tissues regardless of the experimental conditions (Fig. 4G–I), indicating increased glycolytic activity and lactate production in tumors. Furthermore, intermediate metabolites of the glycolytic and fructolytic pathways, such as fructose-6-phosphate (Fig. 4J) and glucose-6-phosphate (Fig. 4K), exhibited varying trends, showing a tendency towards accumulation in tumors.

Notably, the glucose-to-fructose ratio was significantly reduced in tumor tissues (*p* = 0.037) (Fig. 4L), suggesting a potential shift towards enhanced fructose utilization in CRC. These findings collectively indicate a metabolic adaptation in CRC characterized by heightened glycolytic activity, altered fructose metabolism, and a possible reorganization of sugar utilization pathways that may facilitate cancer cell proliferation. This metabolic reprogramming aligns with the Warburg effect, reflecting a preference for glycolytic intermediates in tumor metabolism. The enhanced utilization of fructose in tumor tissues could confer a growth advantage by supporting anabolic processes and maintaining redox balance [11].

## Discussion

This study sheds light on the metabolic reprogramming in CRC, emphasizing the critical role of fructose metabolism. The observed increase in serum D-Fructose and L-Lactic acid following post-glucose load highlights the systemic impact of the PP [12, 13]. The CRC tumor tissue exhibits a significant reduction of the glucose/fructose ratio, which is substantial enough to be reflected in serum metabolite levels. Whilst our findings align with prior research indicating that fructose metabolism enhances glycolysis and supports tumor growth, this is the first evidence of the Warburg effect taking place in CRC patients, with the involvement of the Polyol Pathway; this is consistent with our previous study on AKR1B1-SORD [2, 3].

The evidence for the the Warburg effect in CRC, involving AKR1B1-SORD [2, 3] suggests that this alternative pathway is able to shortcut the regulatory effect of Hexokinase (HK), which is inhibited by its product (G-6-P), as well as phosphofructokinase (PFK), which is inhibited by the ATP steady state levels, see Fig. 1A. Fructolysis, is, thus, able to generate uncontrolled amount of phosphometabolite, fueling lactylation. In addition to this mechanism, the excessive steady state amount of sorbitol, can increase the affinity of π-rich residues and therefore reduce the weakness of π–π and cation-π bonds, hence reducing the affinity of the solvent for charged amino acids and spacers [14]. The resulting changes in the associative electrostatic interactions allow the formation of condensation formations of molecules, lipid droplets that alter stiffness and the proliferation of the tissue [14].

The metabolic heterogeneity observed in CRC highlights the necessity of personalized approaches to treatment. Differences in the activity of metabolic pathways such as the PP could be influenced by tumor genetics, stage, or microenvironmental factors. These variables underline the complexity of targeting metabolic pathways therapeutically. For example, some tumors may rely more heavily on fructose metabolism due to the overexpression of enzymes such as the aldose reductase AKR1B1, while others may depend on alternative pathways for energy production and growth [15].

Importantly, the findings suggest that targeting fructose metabolism could offer a novel stratification approach in CRC. Inhibitors of the PP or key enzymes involved in fructose metabolism, such as the aldose reductase, warrant investigation in preclinical and clinical settings [16]. Combination therapies targeting multiple metabolic pathways may enhance efficacy and overcome resistance mechanisms. For instance, integrating inhibitors of glycolysis with those targeting fructose metabolism could disrupt the metabolic flexibility of CRC cells, forcing them into energy crises and limiting their survival. The implications of targeting fructose metabolism extend beyond CRC. The PP is upregulated in several malignancies, suggesting a broader therapeutic potential. A balance between efficacy and safety must be established through rigorous preclinical testing and biomarker-guided patient selection [17].

Another promising avenue is the integration of metabolic therapies with immune checkpoint inhibitors. Recent studies have shown that metabolic reprogramming in tumors can affect the immune microenvironment. By altering the availability of metabolic intermediates, therapies targeting pathways such as the PP could potentially enhance antitumor immunity. This approach could open new possibilities for combination therapies, particularly for patients who are resistant to existing immunotherapies. Future directions should also include leveraging advanced technologies such as single-cell metabolomics and spatial transcriptomics. These tools can provide unprecedented insights into the metabolic heterogeneity within tumors and their microenvironments. By integrating these approaches with clinical data, researchers can identify subpopulations of CRC patients who are most likely to benefit from metabolic investigation.

Our clinical study highlights the metabolic adaptability of CRC, emphasizing the intricate interplay between glucose and fructose metabolism. The significant increase in serum fructose and lactic acid levels following glucose loading suggests the PP could be a potential therapeutic target, at least to support combination therapies. However, to confirm these findings and facilitate their clinical translation, methodological improvements such as larger sample sizes, optimized sampling intervals, and advanced analytical techniques, are necessary.

## Subjects and methods

### Study design and patient recruitment

A total of 12 patients diagnosed with gastro-intestinal tumors were treated or not with a glucose load prior to surgery. In addition, 17 further samples were collected independently of glucose load. All samples (n=29) underwent molecular analyses (Table 1). Inclusion criteria required histopathological confirmation of CRC and no prior chemotherapy or radiotherapy to minimize confounding factors.

All participants provided informed consent, and the study adhered to ethical guidelines established by the Declaration of Helsinki. Ethical approval was obtained from the institutional review board. Approval was obtained on 09-2019, number 96-19.

### Sample collection

Serum and tissue samples, including tumor and adjacent non-tumorous tissues, were collected during surgical procedures. Serum samples were immediately processed to prevent degradation, while tissue samples were snap-frozen in liquid nitrogen and stored at -80°C until analysis. A detailed clinical history, including comorbidities, medication use, and dietary habits, was documented to contextualize findings [18]. Tumor tissues collection was performed using standardized protocol [19]. All samples were collected within 8 minutes from surgery. Approximately 10 mg of tissue were taken from each sample and processed for molecular gene mutation in the whole genome. The remaining part was fixed in formalin and embedded in paraffin for histological and immunohistochemical analyses. Table 1 reports the principal characteristics of the molecular findings. Hematoxylin and Eosin (H&E) stained serial sections were used for pathological quality control (QC). Histological examination utilized serial sections from formalin-fixed and paraffin-embedded (FFPE) blocks [19]. Two independent pathologists conducted histological analysis on hematoxylin and eosin (H&E)-stained slides. Criteria for selecting tumor samples included a tumor content of at least 30%, necrosis less than or equal to 30%, and the presence of invasive tumor cells. Adjacent normal tissues were also procured. Protein lysate preparation and nucleic acid extraction were carried out using 10 mg of each tissue specimen [20]. Throughout the procedure, tissues remained frozen to maintain integrity.

**Table 1:** Clinical and molecular characteristics.

ID	Tumor site	Age	Gender	Diagnosis	MSI	MMR	Relevant mutations
1	Colon right	70	M	Adenocarcinoma G1 TNM: pT3N0	MSS	/	**KRAS** Gln 61 His (VAF: 39.4%)
2	Colon	78	M	Adenocarcinoma G1 TNM: pT1N0	MSS	/	/
3	Rectum	69	M	Adenocarcinoma G1 TNM: pT3N1a	MSS	/	/
4	Colon right	76	M	Adenocarcinoma G3 TNM: pT3N0	MSS	/	**KRAS** Gly 12 Val (VAF: 43.5%)
5	Colon right	83	W	Adenocarcinoma G3 TNM: pT3N1c	MSS	/	**KRAS** Gly 12 Asp (VAF: 53.2%)
6	Colon right	74	W	Adenocarcinoma G3 TNM: pT4bN1b	MSS	/	**NRAS** Gln 61 Leu (VAF: 37.3%)
7	Colon left	77	W	Adenocarcinoma G3 TNM: pT4aN0	MSS	/	**KRAS** Gly 12 Ala (VAF: 37.5%)
8	Colon - Right	77	W	Adenocarcinoma G1 TNM: pT3N0	MSI-H > 2	**MLH1** and **PMS2**: no expression. **MSH1** and **MSH6**: normally expressed.	/
9	Colon - Left	59	W	Adenocarcinoma G1 TNM: pT3N0	MSS	/	**NRAS** mutation in Q61R
10	Colon left	75	M	Adenocarcinoma G3 TNM: pT3N1c	MSS	/	/
11	Colon left	48	M	Adenocarcinoma G3 TNM: pT4aN1c	MSS	/	**KRAS** Gly 12 Asp (VAF: 47.3%) **DPYD** G2194A: Heterozygote G/A (V732I; c.2194G>A)
12	Colon - Left	74	W	Adenocarcinoma G3 TNM: pT4aN0	MSS	/	**Polymprphism DPYD** G2194A: Mutation G/A - heterozygote
13	Colon - Right	79	W	Adenocarcinoma G3 TNM: pT3N1b	MSS	**MLH1** and **PMS2**: no expression **MSH1** and **MSH6**: normally expressed	**BRAF** mutation in V600E/E complex
14	Colon right	87	W	Adenocarcinoma G3 TNM: pT3N0	MSI-H	**MSH3** Lys 383 frameshift (VAF: 21.7%) **MSH4** Phe 812 frameshift (VAF: 7%)	**BRAF** Val 600 Glu (VAF: 36.4%)
15	Colon right	84	M	Adenocarcinoma G3 TNM: pT3N0	MSI-H	**MSH3** Thr 338 Pro (VAF: 22.4%) **PMS1** Lys 163 frameshift (VAF: 44.7%)	**BRAF** Val 600 Glu (VAF: 40.2%)
16	Ileum	77	M	Adenocarcinoma G3 TNM: pT4aN0	MSS	**MLH3** Leu 267 Arg (VAF: 30.8%) **MSH3** Ser 205 stop (VAF: 9.1%)	**KRAS** Gly 12 Val (VAF: 18.6%)
17	Colon right	81	M	Adenocarcinoma G3 TNM: pT3N0	MSS	/	**KRAS** Gly 12 Val (VAF: 61.6%)
18	Colon right	85	W	Adenocarcinoma G3 p	MSI-H	**EXO1** Thr 479 Ala (VAF: 23.4%) **MSH3** Lys 383 frameshift (VAF: 50%) **PMS2** Asp 414 frameshift (VAF: 19.7%)	/
19	Splenic Flexure	37	W	Adenocarcinoma G3 TNM: pT3N2b	MSS	/	**KRAS** Gly 13 Asp (VAF: 49.1%)
20	Splenic Flexure	73	M	Adenocarcinoma G3 TNM: pT4aN1b	MSS	/	/
21	Colon right	82	W	Adenocarcinoma G3 TNM: pT4aN0	MSI-H	**EXO1** Ile 123 Thr (VAF: 4%) **MLH3** Arg 381 Cys (VAF: 5.4%) **MSH6** Arg 361 His (VAF: 29.4%) **PMS1** Lys 163 frameshift (VAF: 47.8%)	**BRAF** Val 600 Glu (VAF: 22.1%)
22	Sigma - Left	83	M	Adenocarcinoma G1 TNM: pT3N0	MSS	/	/
23	Colon	78	M	Adenocarcinoma G1 TNM pT3N1b	MSS	/	/
24	Sigmoid colon-rectum	85	W	Adenocarcinoma G3 TNM: pT3N0	MSS	/	/
25	Splenic Flexure	55	M	Adenocarcinoma G3 TNM: pT4aN1b	MSS	/	**KRAS** Gly 12 Asp (VAF: 73.4%)
26	Colon right	61	W	Adenocarcinoma G1 TNM: pT3N1a	MSS	**MSH3** Lys 383 frameshift (VAF: 9.8%) **PMS1** Lys 163 frameshift (VAF: 16.1%)	**BRAF** Val 600 Glu (VAF: 12%)
27	Hepatic flexure colon - Right	80	M	Adenocarcinoma G1 TNM: pT3N0	MSS	/	/
28	Recto-sigmoid junction	52	M	Adenocarcinoma G1 TNM: pT3N1(mi)	MSS	**MLH1** and **PMS2**: no expression. **MSH1** and **MSH6**: normally expressed.	**KRAS** mutation in c.38 G > A, p.(Gly13Asp)
29	Liver	74	M	Adenocarcinoma G1 TNM: pT2N0	/	/	/

*MSS* microsatellite stable (no microsatellite instability), *MSI-H* microsatellite instability, *G* grading, *M* man, *W* woman.

### Immunohistochemistry

Paraffin-embedded serial sections were used to evaluate the expression of AKR1B1, SORD and GLUT5 by immunohistochemistry. Briefly, after pre-treatment with citrate buffer Ph8 using a pressure cooker, sections were incubated for 1 h at room temperature with the following primary antibodies: anti-GLUT5 (mouse monoclonal, sc-271055, Santa Cruz Biotechnology); anti-SORD (mouse monoclonal, sc-377200, Santa Cruz Biotechnology); anti-AKR1B1 (rabbit monoclonal, ab316016, Abcam). The reactions have been revealed using the HRP-DAB Detection Kit (UCS Diagnostic, Rome, Italy). The signal was evaluated using Slide Viewer, Quant Center (3DHISTEC, Budapest, Hungary) AI software on digitalized slides (Pannoramic Midi II, Digital Slide Scanner, 3DHISTEC, Budapest, Hungary). The software assessed the positive cells in randomly selected areas. For each sample, the number of positive cells on 0.3 mm^2^ in total have been evaluated.

### Chemicals and reagents

Diisopropylethylamine (DIPEA), 3-nitrophenylhydrazine (3-NPH)·HCl, 1-ethyl-3-(3-dimethylaminopropyl) carbodiimide (EDC) HCl, fructose 1-phosphate,13C6- Fructose and 13C6-Glucose were purchased from Sigma-Aldrich (St Louis, MO, USA). Glucose, fructose, glucose 6-phosphate, serine, glycine, glutamate, glutamine and 2,5-Anhydro-D-mannitol (2,5-AM) were purchased from J&K Scientific Ltd (Beijing, China). LC‑MS‑grade solvents, including methanol (MeOH), acetonitrile (ACN), isopropanol (IPA) and formic acid were purchased from Sigma‑Aldrich. Ultrapure water was prepared by the Millipore purification system (Avidity Science, UK).

### Public database

The related datasets were shown in Supplemental Table I.

The expression levels of GLUT5 (SLC2A5), AKR1B1, and SORD in cancer tissues and normal tissues were analyzed using the Gene Expression Profiles (GEPIA, http://gepia.cancer-pku.cn/) of the Cancer Genome Atlas (TCGA) and Genotype-Tissue Expression (GTEx) databases. Survival analysis of pancreatic cancer tissue samples from the TCGA database using the GEPIA. The correlation between GLUT5 (SLC2A5), SORD and AKR1B1 was analyzed separately using cancer tissue samples from the TCGA database, see Supplemental Table I.

### Expression analysis

#### Spatial transcriptome data

Each microregion in the spatial transcriptome slice is defined by its predominant cell type. For example, when malignant cells comprise the highest proportion in a microregion, the microregion is designated as malignant; when endothelial cells dominate, the microregion is designated as endothelial. The average expression of genes in each cell type per slice was observed, z-score standardized using the scale function, and visualized using the pheatmap package.

#### Single-cell transcriptome data

Gene expression files at the single-cell resolution for CRC were obtained from the TISCH database, and heatmaps of gene expression landscapes were constructed using the pheatmap package. Euclidean distance was used as a metric, and Ward’s method of minimum variance hierarchical clustering facilitated the identification of patterns and trends in the data, aiding in the recognition of conserved gene expression sources.

#### Bulk RNA-seq data

STAR-counts data and corresponding clinical information for 33 tumors were downloaded from the TCGA database (https://portal.gdc.cancer.gov). Data in TPM format were extracted, and normalization was performed using the log2(TPM + 1) transformation. RNA-seq data and corresponding clinical information were selected for further analysis.

### Survival analysis

Kaplan–Meier survival analysis method and R language survival package were used for data analysis. R-packet survminer was used to determine the best cutoff value for SLC2A5, AKR1B1, and SORD gene expression levels to distinguish between high-expression and low-expression groups, ensuring that the proportion of sample size in each group was not less than 0.3. Log-rank test was performed using survfit function to evaluate whether the difference in survival curves between the two groups was statistically significant.

A Cox regression analysis was conducted to assess the association between gene expression and overall survival (OS), disease-free survival (DSS), progression-free interval (PFI), and disease-free interval (DFI). We calculated the Hazard Ratio (HR) and its 95% Confidence Interval (CI) in CRC. Forest plots were used to analyze the association between gene expression and prognosis across various data sets of CRC. The optimal cutoff values for the high-expression and low-expression groups were determined using the survminer package. The log-rank test was performed using the survfit function to evaluate the significance between the high-expression and low-expression groups.

### Correlation analysis between gene expression and tumour cell functional states

The CancerSEA database sorted out the different functional states of 14 tumour cells. The activity of a given pathway was reflected by integrating the expression of characteristic genes, using R package GSVA in z-score parameter calculated the 14 functional state gene sets and obtained the combined z-score. We use the scale function to further standardize the score as the gene set score, and calculate the Pearson correlation between the SLC2A5, AKR1B1, and SORD and each gene set score.

### Single-cell analysis

The single-cell transcriptomic dataset (accession: figshare 25323397) comprising CRC tissue was retrieved in AnnData (H5AD) format. Raw UMI counts and metadata were extracted using anndata (v0.7.5.6) in R 4.4.3, followed by preprocessing through the Seurat v5.2.1 following standard preprocessing workflows. Briefly, the data underwent log-normalization with a scale factor of 10,000 followed by identification of 2000 highly variable genes through variance stabilizing transformation (VST). The data was then scaled and subjected to principal component analysis (PCA) with default parameters. Cell neighborhoods were then determined in PCA space with the first 20 principal components. The data was subsequently embedded into two-dimensional space through Uniform Manifold Approximation and Projection (UMAP) (n.neighbors = 50, min.dist = 0.5, and dims = 1:20). Subsequent analyses were performed exclusively on the tumor cell subset isolated through metadata-based filtering, including cell type verification and expression analyses of the target genes (*AKR1B1*, *SORD* and *SLC2A5*).

### Targeted metabolomics analysis

Metabolite profiling was performed using the Q300 Metabolite Assay Kit (Human Metabolomics Institute, Shenzhen, China) based on previously validated protocols [21]. Key steps included: (i) *Serum sample preparation:* serum samples (20 μL) were mixed with 120 μL of internal standards in a 96-well plate; Following centrifugation at 4000 × *g* for 30 min, supernatants were transferred for derivatization; (ii) *Tissue sample preparation:* tissue samples (20 mg) were homogenized with ultrapure water and internal standards; After centrifugation at 13,500 × *g* and 4 °C for 10 min, supernatants were collected for derivatization; (iii) *Derivatization:* samples were derivatized with reagents at 30 °C for 60 min, dried by lyophilization, and reconstituted in 50% methanol for UPLC-MS analysis [22].

Metabolite concentrations were quantified using UPLC-MS and normalized to internal standards. Statistical analyses included paired t-tests to compare tumor and adjacent tissue metabolites, with significance set at *p* < 0.05. Inter-group variations between glucose-loaded and non-glucose-loaded patients were assessed using ANOVA. Correlation analysis was conducted to explore relationships between metabolite levels and clinical parameters [23]. Moreover, multivariate analysis was performed using Orthogonal Partial Least Squares Discriminant Analysis (OPLS-DA) to identify metabolic features distinguishing glucose-loaded and non-loaded patient groups. The OPLS-DA model was constructed using SIMCA software (v17, Sartorius Stedim Data Analytics AB) with default settings. Model quality was evaluated by the explained variance (R²) and predictive ability (Q²). Variable Importance in Projection (VIP) scores were calculated to determine the contribution of each metabolite to group discrimination. Metabolites with VIP > 1 were considered significant contributors.

Cells were collected and counted, and after washing the cells with cold PBS, the cell precipitate was extracted with 60 μL methanol, then homogenized using a Bullet Blender tissue homogenizer for 3 min and centrifuged at 13,500 × *g* for 10 min at 4 °C. The supernatant was aspirated to spin and 20 μL of freshly prepared derivatization reagent (200 mM 3-NPH in 75% methanol in water and 96 mM EDC-6% pyridine solution in methanol) was added. Derivatization was then performed at 30 °C for 60 min. After derivatization, the samples were lyophilized and then 200 μL of ice-cold 50% methanol solution was added to dissolve the samples. Finally, UPLC-QTOFMS analysis was performed. All analyses were performed on ultra-performance liquid chromatography coupled to a XEVO-G2XS quadrupole time-of-flight mass spectrometry (UPLC-QTOF-MS, Waters Corp., Milford, MA). Chromatographic separation of metabolites was achieved through an Acquity BEH C18 column (100 mm × 2.1 mm i.d.,1.7 μm particle size) equipped with ACQUITY UPLC VanGuard Pre- Column (Waters, Milford, MA, USA) according to the published methods. The column was maintained at 40 °C and a 5 μL aliquot of sample was injected. The flow rate remained constant at 0.3 ml/min. UPLC-MS raw data obtained were analyzed using TargetLynx applications manager version 4.1 (Waters Corp., Milford, MA). Quantification was achieved for each metabolite using linear regression analysis of the peak area of metabolite versus concentration.

### Nucleic acid extraction and quality assessment

Frozen tissue slices were mixed with beta-mercaptoethanol-containing sample buffer and homogenized using the BeadBug system. DNA and RNA were extracted in parallel from the same sample using the Qiagen AllPrep Universal Kit according to the manufacturer’s instructions.

RNA concentration was quantified using Qubit fluorometer with the Qubit RNA BR assay respectively. RNA quality was assessed using the Agilent High-Sensitivity RNA ScreenTape kit respectively. RNAs need to have a RIN ≥ 4 or a DV200 ≥ 60 to be selected for library preparation.

### Library preparation and NGS sequencing

For whole transcriptome sequencing, RNA samples were depleted of the ribosomal RNA using the Ribo Zero Kit (Illumina) and library preparation was performed using the TruSeq Stranded Total RNA Kit (Qiagen). Library preparation kits were used according to manufacturer’s instructions. Sequencing was performed on a NovaSeq6000 system (Illumina). Whole transcriptome sequencing datasets have ≥100 million total reads with less than 20% of ribosomal origin and ≥0 million reads mapping to mRNAs according to Ensembl reference. Ribosomal depletion was performed to remove nuclear rRNA and mt-rRNA.

### Whole proteome sample preparation, measurement and data processing

To align NGS data, Grch38 genome assembly was used as reference. RNA-Seq differential expression was based on normalized readcount data (TPM: transcripts per million).

### Whole proteome sample preparation, measurement and data processing

For whole proteome profiling, 5–10 mg of fresh-frozen tissue was lysed in 2 mL Precellys® CK14 tubes containing 1.4 mm ceramic beads and using a lysis buffer containing PhosSTOP™ and bead shaking using a Precellys® Evolution Homogenizer equipped with a Cryolys® cooling module. After overnight digest samples were acidified and subjected to peptide desalting using Waters HLB Oasis 30 mg 96-well plates. Peptides were desalted using Waters μElution plates, dried down and resolubilized.

For DIA LC–MS/MS measurements, 5 μg of peptides per sample were injected to a reversed phase column (nanoEase M/Z Peptide CSH C18 Column, 1.7 μm, 300 μm × 150 mm) on a Waters ACQUITY UPLC M-Class LC connected to a Thermo Scientific™ Orbitrap Q Exactive™ HF-X mass spectrometer equipped with an EASYspray source. The nonlinear LC gradient was 1 - 60% solvent B in 45 minutes at 50°C and a flow rate of 5 μL/min. The DIA method consisting of one full-range MS1 scan and 50 DIA segments was adapted from Bruderer et al. [24]. Tissue-specific spectral libraries were generated combining high-fractionated DDA and DIA measurements on a pool of tissue material and raw data processed using software DIA-NN version 1.8.1.

## Supplementary information

ST1

S1

S2

## Data Availability

All data are included in the manuscript or available from the corresponding authors upon reasonable request.
