# Supplementary material for: Evidence of fructose metabolism in colorectal cancer
Source: Cell Death Discov. 2025 Oct 16;11:464. doi: 10.1038/s41420-025-02745-w (PMC12533051; doi:10.1038/s41420-025-02745-w)
Supplement: Supplementary file 1 — ST1 [file 41420_2025_2745_MOESM1_ESM.docx]

**Supplemental Table I. List of the related datasets**

| Category | Datasets | Cancer type | Links |
| --- | --- | --- | --- |
| Bulk RNA-seq/Clinical data | TCGA-CRC | Colorectal cancer | https://www.cancer.gov/ccg/research/genome-sequencing/tcga |
| Bulk RNA-seq/Clinical data | GSE87211 | Colorectal cancer | https://www.ncbi.nlm.nih.gov/geo/query/acc.cgi?acc= GSE87211 |
|  | GSE28722 |  | https://www.ncbi.nlm.nih.gov/geo/query/acc.cgi?acc= GSE28722 |
|  | GSE71187 |  | https://www.ncbi.nlm.nih.gov/geo/query/acc.cgi?acc= GSE71187 |
|  | GSE106584 |  | https://www.ncbi.nlm.nih.gov/geo/query/acc.cgi?acc= GSE106584 |
|  | GSE103479 |  | https://www.ncbi.nlm.nih.gov/geo/query/acc.cgi?acc= GSE103479 |
|  | GSE17537 |  | https://www.ncbi.nlm.nih.gov/geo/query/acc.cgi?acc= GSE17537 |
|  | GSE12945 |  | https://www.ncbi.nlm.nih.gov/geo/query/acc.cgi?acc= GSE12945 |
|  | GSE38832 |  | https://www.ncbi.nlm.nih.gov/geo/query/acc.cgi?acc= GSE38832 |
| Spatial transcriptome | CRC1 | Colorectal cancer | https://www.10xgenomics.com/cn/datasets/human-colorectal-cancer-whole-transcriptome-analysis-1-standard-1-2-0 |
|  | CRC2 |  | https://www.10xgenomics.com/cn/datasets/human-intestine-cancer-1-standard |
|  | CRC3 |  | https://aacrjournals.org/cancerdiscovery/article/12/1/134/675646/Spatiotemporal-Immune-Landscape-of-Colorectal |
|  | CRC4 |  | https://aacrjournals.org/cancerdiscovery/article/12/1/134/675646/Spatiotemporal-Immune-Landscape-of-Colorectal |
|  | CRC5 |  | https://aacrjournals.org/cancerdiscovery/article/12/1/134/675646/Spatiotemporal-Immune-Landscape-of-Colorectal |
|  | CRC6 |  | https://aacrjournals.org/cancerdiscovery/article/12/1/134/675646/Spatiotemporal-Immune-Landscape-of-Colorectal |
| Single-cell transcriptome | CRC_EMTAB8107 | Colorectal cancer | https://www.ebi.ac.uk/biostudies/studies/E-MTAB-8107 |
|  | CRC_GSE108989 |  | https://www.ncbi.nlm.nih.gov/geo/query/acc.cgi?acc= GSE108989 |
|  | CRC_GSE112865_mouse_aPD1 |  | https://www.ncbi.nlm.nih.gov/geo/query/acc.cgi?acc= GSE112865 |
|  | CRC_GSE120909_mouse_aPD1 |  | https://www.ncbi.nlm.nih.gov/geo/query/acc.cgi?acc= GSE120909 |
|  | CRC_GSE122969_mouse_aPD1aTIM3 |  | https://www.ncbi.nlm.nih.gov/geo/query/acc.cgi?acc= GSE122969 |
|  | CRC_GSE136394 |  | https://www.ncbi.nlm.nih.gov/geo/query/acc.cgi?acc= GSE136394 |
|  | CRC_GSE139555 |  | https://www.ncbi.nlm.nih.gov/geo/query/acc.cgi?acc= GSE139555 |
|  | CRC_GSE146771_10X |  | https://www.ncbi.nlm.nih.gov/geo/query/acc.cgi?acc= GSE146771 |
|  | CRC_GSE146771_Smartseq2 |  | https://www.ncbi.nlm.nih.gov/geo/query/acc.cgi?acc= GSE146771 |
|  | CRC_GSE166555 |  | https://www.ncbi.nlm.nih.gov/geo/query/acc.cgi?acc= GSE166555 |
|  | CRC_GSE179784 |  | https://www.ncbi.nlm.nih.gov/geo/query/acc.cgi?acc= GSE179784 |
