# Supplementary figures and images for: Evidence of fructose metabolism in colorectal cancer
Source: Cell Death Discov. 2025 Oct 16;11:464. doi: 10.1038/s41420-025-02745-w (PMC12533051; doi:10.1038/s41420-025-02745-w)

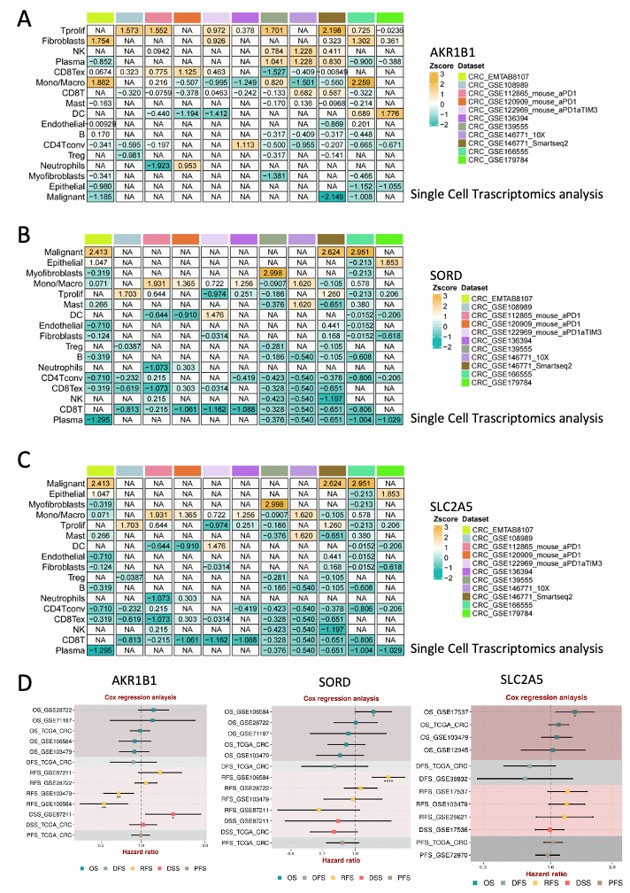

Supplement: Supplementary file 2 — S1 [file 41420_2025_2745_MOESM2_ESM.jpg]

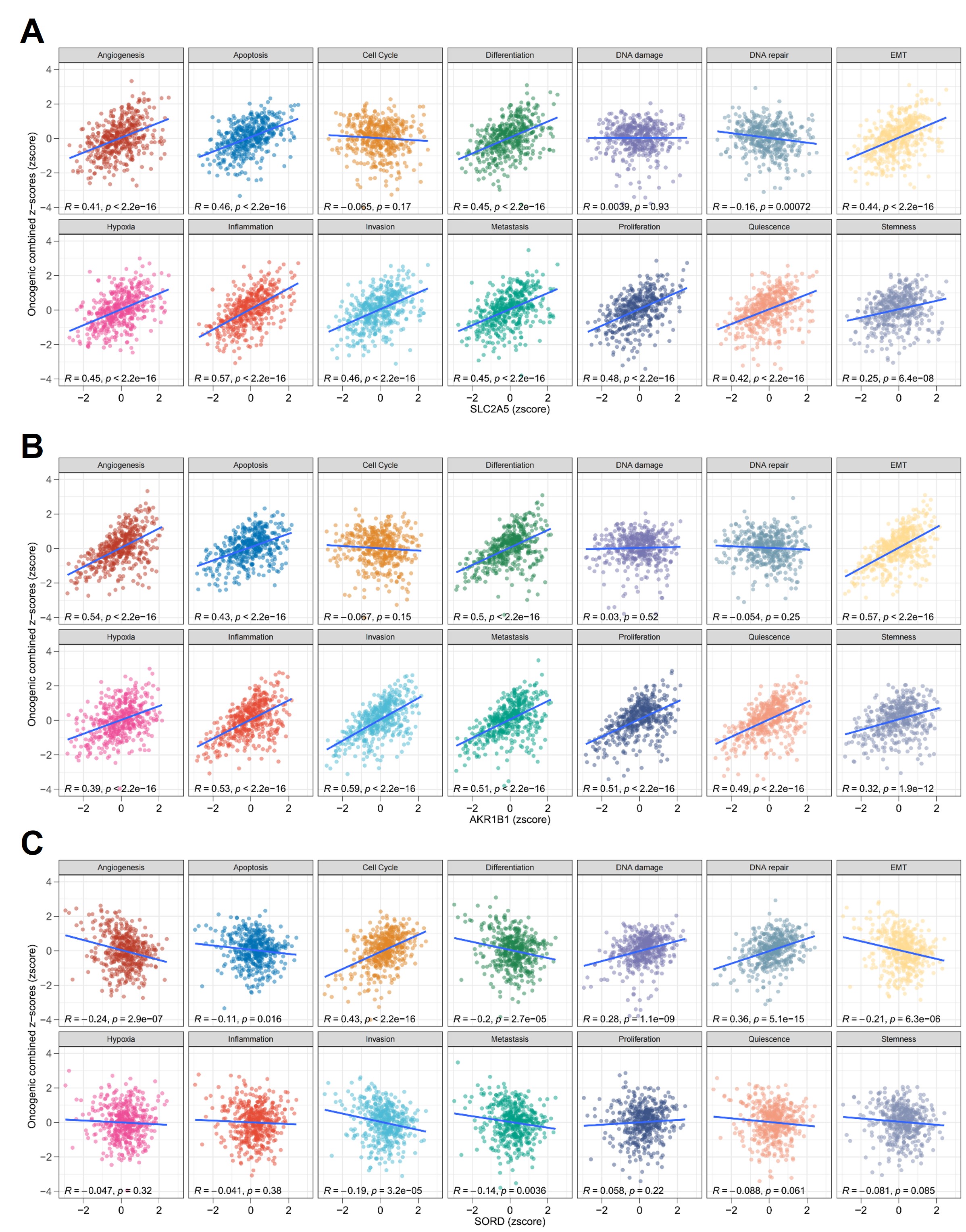

Supplement: Supplementary file 3 — S2 [file 41420_2025_2745_MOESM3_ESM.jpg]
